# Vision expert guided inspection for industrial anomaly detection

**DOI:** 10.1371/journal.pone.0353291

**Published:** 2026-07-08

**Authors:** Xiangyu Zhu, Wenhua Cui, Ye Tao, Xilong Wang

**Affiliations:** 1 School of Electronic and Information Engineering, University of Science and Technology Liaoning, Anshan, China; 2 Key Laboratory of Intelligent Constriguction and Internet of Thinfigs Application Technology, Anshan, China; 3 School of Computer Science and Software Engineering, University of Science and Technology Liaoning, Anshan, China; Polytechnic Institute of Leiria: Instituto Politecnico de Leiria, PORTUGAL

## Abstract

Industrial anomaly detection (IAD), aiming at automatically identifying abnormal patterns that deviate from the normal manufacturing process, plays a critical role in ensuring product quality and equipment safety for intelligent manufacturing systems. In this work, we delve into exploring the generalized and subtle-pattern awarded defect detection. We also propose a visual expert-guided multi-scale anomaly detection method. As the extracted regions often exhibit subtle and vague features that hamper the precise and reliable detection, we leverage the established super-resolution technique to enhance the spatial resolution and recover fine-grained details. It facilitates more discriminative defect representation and improves the model’s capacity at localize anomalies at finer scales. The multi-scale fusion module is constructed by the graph attention network. It aggregates the suspicious regions across different scales by modeling their inter-scale dependencies and contextual relationships. As it dynamically weights and localities those features, it preserves both the micro irregularities and macro structural deviations, hence offering comprehensive anomaly information. Extensive experiments under zero-shot and few-shot settings were conducted on several public datasets. The results demonstrate that the proposed method consistently outperforms existing mainstream approaches in both image-level and pixel-level anomaly detection, achieving pixel-level values of 98.6% and 98.1% under the 4-shot setting on two major benchmarks, and 94.6% under the zero-shot setting, with particularly strong capability in detecting subtle defects on fine-grained textures. It also exhibits enhanced robustness and generalization in cross-domain transfer scenarios.

## 1. Introduction

Industrial anomaly detection represents a critical component in intelligent manufacturing systems [1], aiming to automatically identify abnormal patterns that deviate from normal conditions in manufacturing processes, thereby ensuring product quality and equipment safety. Current research approaches primarily include traditional statistical model-based detection frameworks and end-to-end discriminative methods based on deep learning. In particular, methods leveraging self-supervised learning and generative models have demonstrated considerable effectiveness in constrained scenarios [2]. Nevertheless, the field continues to grapple with several core challenges, a problem primarily due to the extreme scarcity of anomalous samples in industrial settings, which provides insufficient supervisory signals for supervised learning methods and thus severely limits model generalization. Moreover, real industrial anomalies often exhibit subtle deviations in morphology, texture, or structure, especially in high-end manufacturing environments where the differences between normal and anomalous samples are minimal [3]. This subtlety makes it challenging for existing methods to achieve an optimal balance between sensitivity and robustness. In image-based surface defect detection, anomalies frequently manifest as subtle, low-contrast irregularities on product surfaces, occurring across a wide spectrum of industrial products, from high-precision components to conventional hot-rolled steel sheets. Detecting such fine-grained surface defects is a central focus of the present study. The performance of these models is susceptible to significant degradation due to variations in the distribution of data between different production lines, components, or industrial sectors [4]. Similar challenges regarding data quality and environmental interference have also been noted in other engineering domains, such as image degradation in maritime navigation [5] and signal noise in infrastructure monitoring [6]. These challenges underscore the urgent need to develop detection frameworks with stronger generalization capacity and finer discriminative ability.

In recent years, numerous deep learning-based approaches have been developed for industrial anomaly detection. Most studies focus on learning global semantic representations from large-scale normal samples, and then identifying anomalies by comparing test instances against these established normal patterns [7]. However, such methods primarily rely on image-level feature matching. While effective for macroscopic anomalies, this paradigm does not explicitly encourage the model to localize defects, and may therefore fall short in the fine-grained perception of potential defective regions. As for the weak defect scenarios, which often demonstrate vague discriminative features and occupy a subtle areas of the defect regions in the images. those methods struggle to effectively capture the inconspicuous features hence exhibit limited identification capabilities. Several studies, including Anomaly-OV [8] and WinCLIP [9], have attempted to address this issue through multi-scale analysis strategies; however, their effectiveness in fine-grained defect perception is often constrained [10]. Although these methods partition input images into uniform patches through a sliding window mechanism for local inspection, they still suffer from notable drawbacks. Rather than dynamically focusing on semantically suspicious regions, they process the entire image in a rigid mechanical manner. The marginal improvement in sensitivity to subtle anomalies, together with the added computational cost, ultimately hinders their practical deployment in real-time industrial settings.

This paper presents a novel visual expert-guided multi-scale framework designed to advance the field of generalized anomaly detection, with a dedicated focus on identifying subtle structural defects. A primary challenge in this domain stems from the inherently vague and inconspicuous nature of features within localized regions of interest, which complicates reliable diagnostic outcomes. To address this limitation, the proposed method incorporates a super-resolution paradigm for generating magnified image patches. This enhancement of spatial resolution serves to recover fine-grained textual details, thereby fostering a more discriminative representation of potential defects and significantly improving localization precision at finer scales. The framework further integrates a multi-scale fusion module based on a Graph Attention Network. This component is engineered to aggregate features from suspicious regions across different scales by explicitly modeling their inter-dependencies and contextual relationships. Through dynamic attention weighting of these multi-scale features, the model achieves a balanced preservation of both micro-scale irregularities and macro-structural deviations, leading to a comprehensive and robust anomaly assessment.

The method proposed in this study introduces key innovations to existing industrial anomaly detection techniques. The key innovations are listed below:

- We propose a vision expert-guided subtle anomaly enhancement framework for industrial anomaly detection that selectively focuses on semantically suspicious regions rather than performing exhaustive inspection across the entire image, thereby enhancing the detection of weak and fine-grained defects.
- We introduce a region-aware super-resolution enhancement strategy that reconstructs and magnifies suspicious regions, recovering structural and texture details critical for detecting low-resolution and subtle anomalies.
- We design a graph-based multi-scale semantic fusion mechanism that integrates global contextual information with localized anomaly-aware features, enhancing robustness under scale variations and complex industrial scenarios.
- Extensive experiments on MVTec-AD, VisA, and NEU demonstrate that the proposed framework consistently improves zero-shot and few-shot anomaly detection performance and generalizes across different vision expert models.

## 2. Related work

### 2.1 Anomaly detection

Recent years have witnessed rapid progress in deep learning-based methods for industrial visual anomaly detection, with considerable performance achieved on established benchmarks. Current methodologies can be broadly divided into three groups, namely reconstruction-based, feature embedding-based, and synthetic anomaly-based techniques. While these approaches have continuously improved detection performance, the majority still rely on computing anomaly scores across entire image views, lacking dedicated mechanisms for the targeted perception of potentially defective regions.

Reconstruction-based methods typically learn the distribution of normal samples using encoder-decoder architectures and detect anomalies based on reconstruction errors [11–13], For instance, Iqbal et al. [14] proposed a pyramid visual transformer reconstruction network that integrates multi-scale features. Zhang et al. [15] introduced a masked multi-scale reconstruction approach to improve robustness against occlusions and domain shifts, while Hoang et al. [16] combined visual and geometric features for cross-modal anomaly detection. However, these methods often depend on global image reconstruction quality and exhibit limited sensitivity to local subtle anomalies.

Feature embedding-based methods employ pre-trained models to extract image features and identify anomalies by comparing them against normal prototypes stored in a memory bank. For example, Yang et al. [17] improved feature discriminability through self-supervised graph convolution, while Huang W et al. [18] proposed a patch-based deep support vector data description method to capture both structural and detailed information simultaneously. Wang X et al. [19] enhanced sensitivity to small defects via component-level feature augmentation. Although these methods avoid the training overhead associated with reconstruction-based approaches, their anomaly detection relies on global feature distances computed over the entire image or its uniformly partitioned blocks. Consequently, they fail to achieve adaptive focusing on suspicious regions.

The synthetic anomaly method improves the model’s capability to recognize anomalous patterns by generating realistic defective samples to augment the training set. For instance, Peng et al. [20] integrated synthetic anomalies with reconstruction consistency constraints to enhance surface defect detection. Chen et al. [21] proposed a boundary-guided anomaly synthesis strategy to emphasize anomalous features in critical regions, while Zhang et al. [22] developed a comprehensive detection framework combining synthetic data generation, feature selection, and residual analysis. Although synthetic approaches partially mitigate the scarcity of anomalous samples, the synthesis process remains largely based on full images or uniformly divided patches, lacking explicit modeling of potential defective regions.

### 2.2 Zero-/few-shot anomaly detection

In zero-shot and few-shot anomaly detection, several studies have explored cross-class generalization using pre-trained vision-language models [23,24]. For example, Huang C et al. [25] employed a multi-scale memory module to handle complex scenarios, Li et al. [26] adapted CLIP for language-guided anomaly localization, and Jeong et al. [9] leveraged CLIP’s zero-shot capability directly for anomaly detection. Although these methods exhibit certain adaptability under limited sample availability, most still primarily depend on global feature matching over entire images or fixed image patches, without incorporating targeted perception or magnification mechanisms for suspicious regions.

It is worth noting that although some methods employ sliding windows or multi-scale segmentation strategies to improve detection granularity, these approaches typically partition the image in a uniform, grid-like manner. They lack semantic or structural prior guidance to emphasize potentially defective regions. This uniform processing strategy not only incurs substantial computational overhead but may also introduce redundant computation in background areas. Meanwhile, truly subtle anomalies may be diluted during feature aggregation. The limited capacity of existing methods to perceive and localize faint and sparsely distributed anomalies therefore represents an ongoing challenge.

## 3. Method

### 3.1 Overview

**Fig 1:**
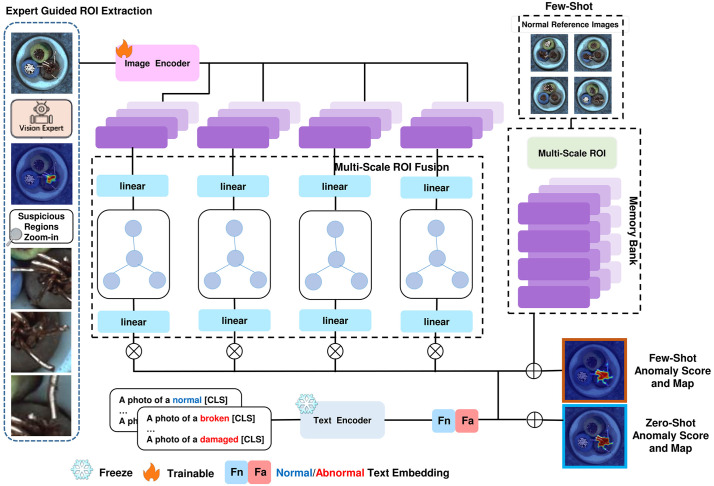
The overview of model structure.

The overall pipeline of the proposed method is illustrated in Fig 1. For a query image $\text{x}\in R^{\text{C}\times \text{H}\times \text{W}}$, the method first utilizes a visual expert model to localize n suspicious regions. The extraction of these regions results in a collection of patches, formally defined as $\left\{x_{\text{1}},x_{\text{2}},\ldots ,x_{n}\right\}$. The method extracts a set of patches $x_{i}\in R^{\text{C}\times H_{i}\times {\text{W}}_{i}}$, where each patch has spatial dimensions $H_{i}<H$ and $W_{i}<W$. These regions are then resized to match the original image dimensions ($\overset{\prime }{\underset{i}{H}}=H$, $\overset{\prime }{\underset{i}{W}}=W$) via a generative super-resolution model. Both the original image and the extracted multi-scale region images are fed into an image encoder to extract multi-scale features. The multi-scale regions are constructed as a star graph where the original image is located at the center node. We propose the ROI multi-scale fusion module constructed by attention graph network to effectively integrate feature information from different sources and scales. Under unsupervised training, the image features from the encoder, together with the text features, are input into the multi-scale fusion module to produce an image-level anomaly score S and a pixel-level anomaly localization map $\text{M}\in R^{H\times W}$. In the few-shot setting, the system localizes and detects anomalies by comparing image patches against features of normal samples stored in a memory bank.

### 3.2 Vision expert guided inspection

#### 3.2.1 Region-of-interest patches extraction.

In industrial anomaly detection, partitioning entire images into patches typically incurs high computational overhead and redundant operations, primarily due to the fact that most patches contain no meaningful defect information. To address this limitation, this paper introduces a visual expert-guided region of interest extraction strategy. The approach leverages existing well-established anomaly detection models, including WinCLIP [9], AdaCLIP [27], and AA-CLIP [28], as vision experts to produce initial anomaly response maps. Regions with anomaly scores exceeding a threshold of 0.3 are selected as candidate anomalous zones to ensure broad coverage of potential defect locations.

To further aggregate discrete anomaly response points, the K-means algorithm is applied to cluster these hotspots into K salient regions. Bounding boxes are then computed for each cluster to obtain preliminary localization of key areas. To enhance contextual integrity, each bounding box is slightly expanded and resized to several fixed scales, including 64 × 64, 128 × 128, and 256 × 256 pixels. Each extracted region is then placed onto a zero-background anchor matrix matching the original image size, forming a region representation $A_{i}=x_{i}\cup \text{0}\in R^{C\times H\times W}$ with explicit positional information. This representation preserves both the local characteristics of the suspicious region and its spatial location within the global image.

#### 3.2.2 Enhancement with super resolution.

Since the extracted suspicious regions are considerably smaller than the original image, directly analyzing these areas often fails to capture effective defect-related features. To address this issue, this paper introduces a magnification strategy based on a generative super-resolution model, which aims to restore the small suspicious regions to the original image resolution, thereby enhancing the characterization of defects. For the super-resolution reconstruction, the Stable Diffusion model [29] is leveraged to augment the spatial resolution and recover fine-grained details in the suspicious regions. This type of model synthesizes visually faithful image details through an iterative denoising process and has demonstrated strong performance in image super-resolution tasks. Through this process, the suspicious regions are transformed into a set of magnified image patches $\left\{I_{\text{1}},I_{\text{2}},\ldots ,I_{n}\right\}$, where each $\;I_{i}\in R^{C\times H\times W}$. This process significantly improves the visibility of defect features while preserving the semantic information of the regions.

### 3.3 Multi-scale region-of-interest fusion

**Fig 2:**
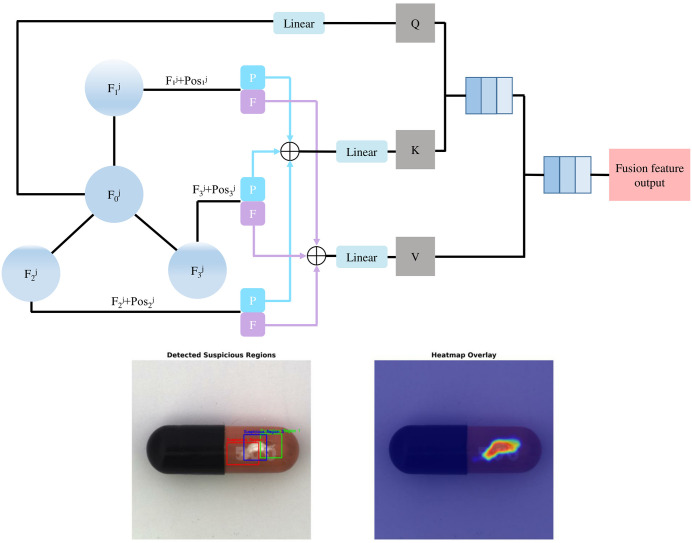
A multi-scale feature fusion framework leveraging star graph attention mechanism.

This graph attention-based fusion mechanism exhibits three key characteristics. It preserves the hierarchical structure of features, enabling organic integration of visual information across different scales. By employing spatial-semantic dual attention, the model enhances its sensitivity to subtle anomalous patterns. The messaging mechanism of the star graph further ensures computational efficiency throughout the feature fusion process. Ultimately, the fusion module produces a fused block feature for each layer. As shown in Fig 2, this design enables the selective enhancement of anomaly-related local features while maintaining a consistent global context, which ultimately yields a more robust feature set for anomaly scoring and localization.

During the feature extraction stage, this work adopts a typical processing pipeline similar to methods such as WinCLIP and AdaCLIP. The image encoder is divided into four hierarchical levels, and patch-level features are extracted from the intermediate layers of each level. Both the original image and all images of the suspicious regions are independently fed into the encoder to obtain their corresponding multi-scale features. Additionally, the original unmagnified anchor images of the suspicious regions are also encoded to extract spatial embedding features that represent their positional and extent information. This process can be formally described as follows:


$$\begin{array}{c} \left[\overset{\text{0}}{\underset{i}{F}},\overset{\text{1}}{\underset{i}{F}},\overset{\text{2}}{\underset{i}{F}},\overset{\text{3}}{\underset{i}{F}}\right]=Encoder(I_{i}) \end{array}$$ (1)

$$\begin{array}{c} \left[\overset{\text{0}}{\underset{i}{Pos}},\overset{\text{1}}{\underset{i}{Pos}},\overset{\text{2}}{\underset{i}{Pos}},\overset{\text{3}}{\underset{i}{Pos}}\right]=Encoder(A_{i}) \end{array}$$ (2)

Where I denotes the collective of original image with i=0 and suspicious regions i>0. $\overset{j}{\underset{i}{F}}$ denotes the patch feature at the stage j, and $\overset{j}{\underset{i}{Pos}}$ denotes the positional embedding at the stage j.

To integrate multi-scale patch features, a star graph structure is constructed with the original image features at the center node and features from various suspicious regions as surrounding nodes. Feature interaction is achieved through a message-passing mechanism. Within this graph, an attention-based fusion method is designed. Specifically, at each level j, the original image feature $\overset{j}{\underset{\text{0}}{F}}$ is formulated by Equation 3. The query vector encapsulates the semantic context of the global image and functions as a baseline reference for the selection and fusion of features.


$$\begin{array}{c} Q^{j}=linear(\overset{j}{\underset{\text{0}}{F}}) \end{array}$$ (3)

After concatenating all embedded features, the resulting representation is linearly transformed into the key vector $K^{j}$, as expressed in Equation 4. This key vector captures the spatial distribution of each local region along with its relative positional relationship to the global image. Hence, it offers a searchable spatial-semantic index for subsequent query matching.


$$\begin{array}{c} K^{j}=linear(Concat\left(\left[\overset{j}{\underset{\text{0}}{Pos}},\overset{j}{\underset{\text{1}}{Pos}},\ldots ,\overset{j}{\underset{k}{Pos}}\right]\right)) \end{array}$$ (4)

Upon concatenation, all image patch features are linearly transformed into the value vector $V^{j}$, as specified in Equation 5. The value vector aggregates multi-scale visual features derived from both global and local regions, thereby constituting the foundational information source for the fusion process.


$$\begin{array}{c} V^{j}=linear(Concat\left(\left[\overset{j}{\underset{\text{0}}{F}},\overset{j}{\underset{\text{1}}{F}},\ldots ,\overset{j}{\underset{k}{F}}\right]\right)) \end{array}$$ (5)

The attention weight is computed by scaling the dot product. This process constructs a correlation matrix between the global semantic context and local spatial locations, thereby quantifying the relative contribution of each local region to the global feature representation. The resulting fusion feature is given by Equation 6, where d denotes the feature dimension.


$$\begin{array}{c} F^{j}=linear(\left(softmax\left(\frac{{Q^{j}\left(K^{j}\right)}^{T}}{\sqrt{d}}\right)\right)V^{j}) \end{array}$$ (6)

This mechanism effectively recalibrates features across different regions and scales in both semantic and spatial dimensions, allowing the central node to adaptively incorporate the most anomaly-relevant local contextual information. Ultimately, the fusion module outputs a fused patch feature for each level, enriched with multi-scale context from both the original image and all suspicious regions.

### 3.4 Zero-shot & Few-shot

In the zero-shot anomaly detection setting, the proposed method computes the anomaly score by comparing the multi-scale fused region-of-interest patch features against predefined text features. Specifically, the feature maps from all four levels are upsampled, and their similarity to the text embeddings is computed. The pixel-level anomaly segmentation result is obtained as:


$$\begin{array}{c} \overset{zero}{\underset{seg}{S}}=\frac{\text{1}}{\text{4}}\sum_{l=\text{1}}^{\text{4}}Up(softmax(F_{l}{\left(Text\;Emb\right)}^{T})) \end{array}$$ (7)

$$\begin{array}{c} \overset{zero}{\underset{cls}{S}}=\frac{\text{1}}{\text{4}}\sum_{l=\text{1}}^{\text{4}}\underset{patches}{\text{max}}(softmax(F_{l}{\left(Text\;Emb\right)}^{T})) \end{array}$$ (8)

Where $\overset{zero}{\underset{seg}{S}}$ denots the zero-shot anomaly segmentation results. The anomaly maps are upsampled to the same resolution to the original image. $\overset{zero}{\underset{cls}{S}}$ is the zero-shot anomaly classification results.

In the few-shot setting, the system leverages the provided normal samples to extract multi-level features through the vision expert-guided region-of-interest (ROI) extraction module and the multi-scale fusion module. These features are used to construct a memory bank of normal features. During inference, the system generates a supervised anomaly segmentation map $\overset{sup}{\underset{seg}{S}}$ and a classification score $\overset{sup}{\underset{cls}{S}}$ by computing the cosine distance between the features of the query image and those stored in the normal feature memory.

To further enhance detection performance, this paper introduces a weighted fusion strategy that combines the results from both zero-shot and few-shot detection. The fused results are defined as:


$$\begin{array}{c} \overset{few}{\underset{seg}{S}}=\text{0}.\text{5}\times \overset{zero}{\underset{seg}{S}}+\text{0}.\text{5}\times \overset{sup}{\underset{seg}{S}} \end{array}$$ (9)

$$\begin{array}{c} \overset{few}{\underset{cls}{S}}=\text{0}.\text{5}\times \overset{zero}{\underset{cls}{S}}+\text{0}.\text{5}\times \overset{sup}{\underset{cls}{S}} \end{array}$$ (10)

This fusion mechanism leverages both the generalization capability of zero-shot detection and the domain adaptability of few-shot learning, thereby achieving more robust anomaly detection across diverse real-world scenarios.

### 3.5 Loss function

To achieve precise localization of pixel-level anomalous regions, the model is jointly optimized using a multi-objective loss function comprising cross-entropy loss, focal loss, and Dice loss. The cross-entropy loss defined as:


$$\begin{array}{c} L_{ce}=-\sum_{i=\text{1}}^{n}y_{i}\;\text{log}\left(p_{i}\right) \end{array}$$ (11)

Measures the discrepancy between the true pixel label $y_{i}$ and the predicted probability $p_{i}$. It is widely used in semantic segmentation tasks to enhance inter-class discriminability. To address class imbalance, the focal loss is introduced.


$$\begin{array}{c} L_{focal}=-\frac{\text{1}}{n}\sum_{i=\text{1}}^{n}{\left(\text{1}-p_{i}\right)}^{\gamma }\text{log}\left(p_{i}\right) \end{array}$$ (12)

This loss reduces the influence of easily classified examples through the modulating factor ${\left(\text{1}-p_{i}\right)}^{\gamma }$, thus directing greater attention to challenging and rare anomalous regions. Additionally, to improve boundary fitting quality and spatial consistency, the Dice loss is employed:


$$\begin{array}{c} L_{dice}=-\frac{\sum_{i=\text{1}}^{n}y_{i}{\hat{y}}_{i}}{\sum_{i=\text{1}}^{n}\overset{\text{2}}{\underset{i}{y}}+\sum_{i=\text{1}}^{n}\overset{\text{2}}{\underset{i}{\hat{y}}}} \end{array}$$ (13)

This quantifies the overlap between the prediction ${\hat{y}}_{i}$ and the ground truth $y_{i}$, thereby increasing the model’s sensitivity to the shape and location of anomalous areas. The overall loss function is a weighted sum of the three components:


$$\begin{array}{c} \text{L}=\alpha L_{ce}+\beta L_{focal}+\delta L_{dice} \end{array}$$ (14)

Where α, β, and δ are hyper parameters that balance the contributions of each loss term during training, through multi-objective optimization, the model demonstrates enhanced robustness and higher accuracy in pixel-level anomaly localization.

## 4. Experiments

### 4.1 Datasets

The proposed method was comprehensively evaluated on three industrial anomaly detection datasets, including the widely adopted public benchmarks MVTec Anomaly Detection (MVTec-AD) [30] and Visual Anomaly (VisA) [31], as well as a real-world dataset from the metallurgy industry, the Northeastern University (NEU) surface defect database [32]. All models were trained using only the normal samples from each dataset to adhere to real-world industrial anomaly detection conditions.

MVTec-AD comprises 15 categories of industrial products, with a training set of 3,629 normal images and a test set of 1,725 images containing various defect types and pixel-level annotations. VisA presents a more challenging benchmark with 12 object categories, featuring greater intra-class variation and more complex backgrounds. It includes 8,659 normal training images and 2,162 test images, of which 1,200 contain anomalies. The NEU surface defect database was adopted to validate performance in a metallurgical industrial context, containing 1,800 grayscale images across six typical hot-rolled steel strip surface defects.

To rigorously assess the model’s capability in detecting subtle defects, a multi-scale evaluation setup was established by proportionally resizing the images to 50% and 25% of their original dimensions, creating low-contrast, challenging detection scenarios.

### 4.2 Evaluation metrics

To evaluate the performance of the proposed method in industrial anomaly detection, we adopted image-level Area Under the Receiver Operating Characteristic Curve (AUROC) and pixel-level AUROC as the primary metrics. These criteria provide an objective assessment of the model’s capability in both anomaly classification and localization.

Image-level AUROC evaluates the model’s ability to distinguish between normal and abnormal images. This metric is computed as the area under the curve plotting the true positive rate (TPR) against the false positive rate (FPR) across various classification thresholds. A value closer to 1 indicates superior overall classification performance.

Pixel-level AUROC, on the other hand, assesses the accuracy of anomaly localization. It is derived by comparing pixel-wise predictions with ground truth annotations and calculating the area under the corresponding ROC. This metric effectively reflects the model’s capability in detecting subtle defects and delineating their boundaries.

### 4.3 Base models

The proposed method was evaluated against several representative baselines in industrial anomaly detection. WinCLIP [9] performs zero-shot and few-shot anomaly classification and segmentation by combining state words with prompt templates across window, patch, and image-level features. Similarly, AdaCLIP [27] introduces learnable static and dynamic prompts and is further trained on auxiliary data to improve cross-category generalization. In contrast, PatchCore [33] constructs a memory bank of multi-scale normal features for high-precision anomaly localization via similarity matching. SPADE [34] generates anomaly score maps using nearest-neighbor search in deep feature space, making it suitable for few-shot scenarios. APRIL-GAN [35] employs a hybrid architecture that integrates generative adversarial networks with feature matching in a joint image-text embedding space, enhanced by multiple memory banks under few-shot settings. PromptAD [36] introduces a one-class prompt learning framework that transforms normal prompts into anomalous versions via semantic splicing to construct negative samples and guide model training. Finally, AA-CLIP [28] addresses the inherent anomaly unawareness of CLIP through a two-stage adaptation strategy that disentangles anomaly-aware text anchors and aligns them with patch-level visual features, incorporating lightweight residual adapters to retain generalization while boosting anomaly sensitivity.

These baseline models encompass a diverse range of technical approaches, including feature reconstruction, memory-based mechanisms, prompt learning, and large-scale model inference, providing a representative and comprehensive benchmark for this study.

### 4.4 Implementation details

All experiments were conducted on a server equipped with an NVIDIA GeForce RTX 4090 GPU (24 GB), an Intel Xeon Silver 4314 CPU (64 cores), and 125 GB of RAM, running Ubuntu 24.04 LTS (kernel 6.17.0). The software environment included Python 3.12.0, PyTorch 2.10.0 with CUDA 12.8, Diffusers 0.21.0, Transformers 4.36.0, and OpenAI CLIP 1.0. The super-resolution module employed the Stable Diffusion x4 upscaler (stabilityai/stable-diffusion-x4-upscaler, checkpoint x4-upscaler-ema.ckpt), performing 4 × upscaling with 75 denoising steps using pretrained weights without fine-tuning on industrial textures. Average inference time for a 1024 × 1024 image was 3.5 s, with 2.8 s consumed by the Stable Diffusion upscaling, and peak GPU memory usage of 8.5 GB. During few-shot training, the CLIP vision and text encoders were frozen, while only the learnable prompt tokens and the multi-scale fusion module were optimized, with loss function hyperparameters set to α = 0.7, β = 0.3, and δ = 0.5. For visual expert-guided ROI extraction, candidate anomalous regions were defined as areas with anomaly scores exceeding 0.3, a threshold empirically determined on a validation subset of MVTec AD to balance defect coverage and false positives. These candidate points were subsequently grouped into coherent suspicious regions using K-means clustering with k = 3.

## 5. Experimental results

### 5.1 Comparison on few-shot anomaly detection

As shown in Table 1, the proposed method was systematically compared with existing mainstream baseline models under few-shot anomaly detection settings. Evaluation metrics included image-level AUROC [37] and pixel-level AUROC. Experiments were conducted on the MVTec-AD, VisA, and NEU surface defect database, covering various few-shot configurations including 1-, 2-, and 4-shot scenarios.

**Table 1:** Few-shot IAD results on MVTec-AD, VisA, and NEU surface defect database.

Setup		MVTec-AD		VisA		NEU	
		Image-AUC	Pixel-AUC	Image-AUC	Pixel-AUC	Image-AUC	Pixel-AUC
1-shot	WinCLIP	93.1 ± 2.0	95.2 ± 0.5	83.8 ± 4.0	96.4 ± 0.4	82.5 ± 3.5	94.8 ± 0.6
	AdaCLIP	89.2 ± 1.8	88.7 ± 1.2	85.8 ± 3.5	95.5 ± 0.6	84.3 ± 3.2	94.2 ± 0.8
	PatchCore	83.4 ± 3.0	92.0 ± 1.0	79.9 ± 2.9	95.4 ± 0.6	78.6 ± 3.1	93.5 ± 0.9
	SPADE	81.0 ± 2.0	91.2 ± 0.4	79.5 ± 4.0	95.6 ± 0.4	77.8 ± 3.8	92.8 ± 0.7
	APRIL-GAN	92.0 ± 0.3	95.1 ± 0.1	91.2 ± 0.8	96.0 ± 0.0	90.5 ± 1.2	95.3 ± 0.3
	PromptAD	94.6 ± 1.7	95.9 ± 0.5	86.9 ± 2.3	96.7 ± 0.4	85.7 ± 2.5	95.1 ± 0.5
	AA-CLIP	94.4 ± 1.3	96.0 ± 0.3	84.6 ± 2.4	96.8 ± 0.3	83.8 ± 2.1	94.9 ± 0.4
	**ours**	95.8 ± 1.0	97.8 ± 0.2	92.6 ± 1.0	96.5 ± 0.2	91.8 ± 1.0	95.8 ± 0.2
2-shot	WinCLIP	94.4 ± 1.3	96.0 ± 0.3	84.6 ± 2.4	96.8 ± 0.3	83.8 ± 2.1	95.5 ± 0.4
	AdaCLIP	90.8 ± 1.6	89.5 ± 1.0	86.9 ± 3.0	96.0 ± 0.5	85.7 ± 2.8	95.1 ± 0.6
	PatchCore	86.3 ± 3.3	93.3 ± 0.6	81.6 ± 4.0	96.1 ± 0.5	80.4 ± 3.5	94.6 ± 0.7
	SPADE	82.9 ± 2.6	92.0 ± 0.3	80.7 ± 5.0	96.2 ± 0.4	79.3 ± 4.2	93.9 ± 0.5
	APRIL-GAN	92.4 ± 0.3	95.5 ± 0.0	92.2 ± 0.3	96.2 ± 0.0	91.3 ± 0.9	95.8 ± 0.2
	PromptAD	95.7 ± 1.5	96.2 ± 0.3	88.3 ± 2.0	97.1 ± 0.3	87.2 ± 1.8	96.3 ± 0.4
	AA-CLIP	95.2 ± 1.3	96.2 ± 0.3	87.3 ± 1.8	97.2 ± 0.2	85.9 ± 1.5	95.7 ± 0.3
	**ours**	96.0 ± 0.8	98.2 ± 0.2	93.3 ± 0.8	97.8 ± 0.2	92.5 ± 0.8	96.6 ± 0.2
4-shot	WinCLIP	95.2 ± 1.3	96.2 ± 0.3	87.3 ± 1.8	97.2 ± 0.2	85.9 ± 1.5	96.1 ± 0.3
	AdaCLIP	91.9 ± 1.4	90.1 ± 0.8	88.2 ± 2.5	96.4 ± 0.4	87.4 ± 2.3	95.8 ± 0.5
	PatchCore	88.8 ± 2.6	94.3 ± 0.5	85.3 ± 2.1	96.8 ± 0.3	83.7 ± 2.3	95.4 ± 0.6
	SPADE	84.8 ± 2.5	92.7 ± 0.3	81.7 ± 3.4	96.6 ± 0.3	80.9 ± 3.1	94.5 ± 0.4
	APRIL-GAN	92.8 ± 0.2	95.9 ± 0.0	92.6 ± 0.4	96.2 ± 0.0	92.0 ± 0.7	96.1 ± 0.1
	PromptAD	96.6 ± 0.9	96.5 ± 0.2	89.1 ± 1.7	97.4 ± 0.3	88.5 ± 1.4	96.8 ± 0.3
	AA-CLIP	96.5 ± 0.9	96.5 ± 0.2	89.1 ± 1.7	97.4 ± 0.3	88.5 ± 1.4	96.3 ± 0.2
	**ours**	97.8 ± 0.5	98.6 ± 0.1	94.8 ± 0.5	98.1 ± 0.1	93.2 ± 0.5	97.2 ± 0.1

In terms of overall performance, the proposed method consistently outperforms all baseline models across all datasets and few-shot settings, demonstrating leading results in both image-level and pixel-level anomaly detection metrics. Specifically, under the one-shot setting, the method achieves image-level AUROC scores of 95.8% on MVTec-AD and 92.6% on VisA, improving upon the previous best models by 1.2 to 5.8 percentage points. It also attains an image-level AUROC of 91.8% on the NEU metal defect dataset. For pixel-level localization, the method reaches an AUROC of 97.8% on MVTec-AD, significantly surpassing other models. When the sample size increases to four, the image-level AUROC further improves to 97.8% on MVTec-AD and 94.8% on VisA, while the pixel-level AUROC values reach 98.6% and 98.1%, maintaining a clear advantage.

In comparison, baseline models such as WinCLIP and AA-CLIP perform competitively in image-level classification but show limitations in localizing subtle defects. Although PatchCore and SPADE exhibit acceptable localization performance in certain categories, their results depend heavily on the quantity and quality of normal samples. Prompt learning-based approaches, including AdaCLIP and PromptAD, demonstrate unstable performance in cross-category generalization. These experimental results confirm that the proposed method offers stronger adaptability and stability in complex industrial scenarios.

**Fig 3:**
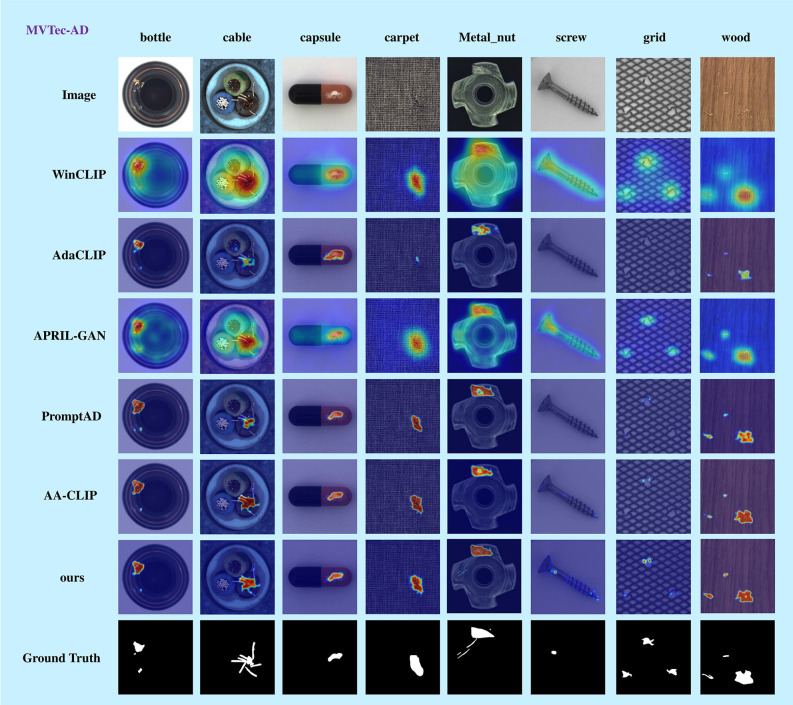
Visualization detection results for MVtec-AD.

**Fig 4:**
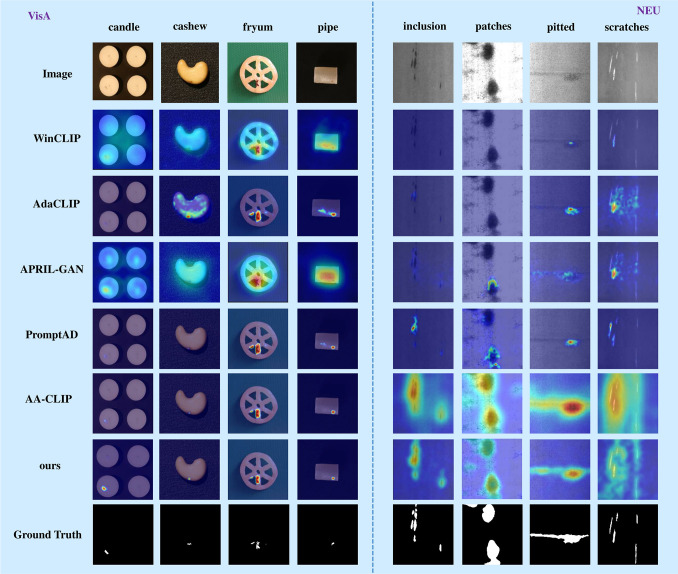
Visualization detection results for Visa and NEU datasets.

These results demonstrate the effectiveness of the proposed multi-scale visual expert mechanism and region fusion strategy under few-shot settings. The performance advantage primarily stems from the precise localization of suspicious regions and the effective integration of multi-scale contextual information, enabling more stable and refined anomaly identification with limited samples. As illustrated in Figs 3 and 4, the visualization results further confirm that the proposed method achieves superior defect boundary clarity and response consistency compared to other models, while maintaining high classification accuracy.

### 5.2 Comparison on zero-shot anomaly detection

Under the zero-shot anomaly detection setting, quantitative comparison results between the proposed method and existing mainstream models are presented in Table 2. Empirical results on multiple benchmarks, including MVTec-AD, VisA, and the NEU dataset, confirm that the proposed method consistently outperforms all baseline models in both image-level and pixel-level evaluation, underscoring its strong generalization and cross-domain stability.

**Table 2:** Zero-shot anomaly detection results.

Model	Industrial Defects		
	MVTec-AD	VisA	NEU
WinCLIP	91.8	78.8	82.5
APRIL-GAN	86.2	78.0	80.8
AdaCLIP	89.2	85.8	84.3
Anomaly-OV	94.0	91.1	89.7
**ours**	94.6	92.3	90.5

It is worth noting that the construction of text features plays a critical role in the performance of zero-shot anomaly detection. For instance, WinCLIP relies on manually designed text prompt templates, which can maintain discriminative power in certain categories. However, owing to the limited semantic coverage of the predefined prompts, it often fails to capture the diverse anomalous patterns present across different industrial scenarios, leading to constrained detection performance. In contrast, the proposed method leverages a pre-trained vision-language model to automatically extract semantic features aligned with image content, thereby mitigating bias introduced by manual design. This approach results in more robust alignment between textual and visual representations.

### 5.3 Comparison on subtle defect detection

In the image down-scaling experiment designed to simulate subtle defect detection, a comparison of the proposed method with other approaches is presented in Tables 3 and 4. This experiment evaluates the robustness of the models under low-resolution conditions by down-scaling the original images to create more challenging detection scenarios with less discernible defects. The results demonstrate that the proposed method consistently outperforms existing state-of-the-art models in both image-level and pixel-level AUROC across various scaling factors, demonstrating its consistent and stable capability in detecting subtle anomalies.

**Table 3:** Performance comparison on subtle defect detection at 50% scaling.

Setup		MVTec-AD		VisA		NEU	
		Image-AUC	Pixel-AUC	Image-AUC	Pixel-AUC	Image-AUC	Pixel-AUC
1-shot	WinCLIP	90.2 ± 2.3	90.3 ± 2.0	80.9 ± 3.1	91.5 ± 2.5	79.6 ± 3.0	89.9 ± 2.8
	AdaCLIP	86.3 ± 3.0	83.8 ± 3.7	82.9 ± 4.0	90.6 ± 3.2	81.4 ± 3.5	89.3 ± 3.3
	AA-CLIP	92.2 ± 1.8	91.4 ± 1.5	85.5 ± 2.6	92.3 ± 2.0	84.3 ± 2.4	91.0 ± 2.2
	**ours**	94.8 ± 1.0	95.5 ± 0.7	90.2 ± 1.5	94.6 ± 1.1	89.6 ± 1.8	93.8 ± 1.3
2-shot	WinCLIP	91.5 ± 2.0	91.1 ± 1.8	81.7 ± 3.0	91.9 ± 2.2	80.9 ± 2.8	90.6 ± 2.5
	AdaCLIP	87.9 ± 2.5	84.6 ± 3.0	84.0 ± 3.7	91.1 ± 2.8	82.8 ± 3.0	80.2 ± 3.1
	AA-CLIP	93.6 ± 1.5	91.7 ± 1.2	86.7 ± 2.2	92.5 ± 2.5	85.9 ± 2.0	91.8 ± 1.8
	**ours**	95.5 ± 0.8	96.2 ± 0.6	91.1 ± 1.3	95.3 ± 0.8	90.7 ± 1.4	94.6 ± 1.1
4-shot	WinCLIP	92.3 ± 1.5	91.3 ± 1.3	84.4 ± 2.4	92.3 ± 2.0	83.0 ± 2.7	91.2 ± 2.2
	AdaCLIP	89.0 ± 2.1	85.2 ± 2.5	85.3 ± 3.3	91.5 ± 2.5	84.5 ± 2.8	90.9 ± 2.8
	AA-CLIP	94.4 ± 1.2	92.3 ± 1.0	88.7 ± 1.8	92.8 ± 1.6	87.4 ± 1.8	92.4 ± 1.5
	**ours**	96.3 ± 0.5	96.8 ± 0.3	91.7 ± 1.0	96.3 ± 0.4	91.1 ± 1.1	95.2 ± 0.7

**Table 4:** Performance comparison on subtle defect detection at 25% scaling.

Setup		MVTec-AD		VisA		NEU	
		Image-AUC	Pixel-AUC	Image-AUC	Pixel-AUC	Image-AUC	Pixel-AUC
1-shot	WinCLIP	87.2 ± 3.7	85.3 ± 2.9	77.9 ± 4.4	86.5 ± 3.5	76.6 ± 4.1	84.9 ± 3.8
	AdaCLIP	83.3 ± 3.8	78.8 ± 4.3	79.9 ± 5.2	85.6 ± 4.0	78.4 ± 4.5	84.3 ± 3.7
	AA-CLIP	90.2 ± 2.5	87.4 ± 2.2	83.5 ± 3.5	88.3 ± 3.0	82.3 ± 3.5	87.0 ± 3.2
	**ours**	92.3 ± 1.5	92.8 ± 1.2	87.7 ± 2.0	91.5 ± 1.8	86.8 ± 2.5	90.5 ± 2.0
2-shot	WinCLIP	88.5 ± 3.0	86.1 ± 2.7	78.7 ± 3.6	86.9 ± 3.2	77.9 ± 4.2	85.6 ± 3.5
	AdaCLIP	84.9 ± 3.5	79.6 ± 4.0	81.0 ± 4.3	86.1 ± 3.8	79.8 ± 3.9	75.2 ± 4.5
	AA-CLIP	91.6 ± 1.9	87.7 ± 1.8	84.7 ± 2.9	88.5 ± 3.3	83.9 ± 3.1	87.8 ± 2.8
	**ours**	93.3 ± 1.2	93.6 ± 1.0	88.2 ± 1.8	92.2 ± 1.5	87.5 ± 2.0	91.3 ± 1.8
4-shot	WinCLIP	89.3 ± 2.5	86.3 ± 2.1	81.4 ± 3.6	87.3 ± 3.0	80.0 ± 3.5	86.2 ± 3.2
	AdaCLIP	86.0 ± 3.0	80.2 ± 3.4	82.3 ± 4.0	86.5 ± 3.1	81.5 ± 3.6	85.9 ± 3.8
	AA-CLIP	93.4 ± 1.6	88.3 ± 1.5	86.7 ± 2.4	88.8 ± 2.3	85.4 ± 2.7	88.4 ± 2.5
	**ours**	94.0 ± 0.8	94.3 ± 0.6	89.2 ± 1.5	93.1 ± 1.1	88.5 ± 1.9	92.3 ± 1.2

As the image size decreases, the performance of most comparison models declines significantly. This effect is particularly evident in methods relying on global feature matching, such as PatchCore and WinCLIP. Due to the loss of fine-grained details and degraded feature representation, these models struggle to extract discriminative defect features from severely degraded images. In contrast, the proposed method actively focuses on suspected regions and enhances them locally via a vision expert-guided region-extraction mechanism coupled with generative super-resolution magnification. This approach effectively mitigates information loss due to reduced resolution.

**Fig 5:**
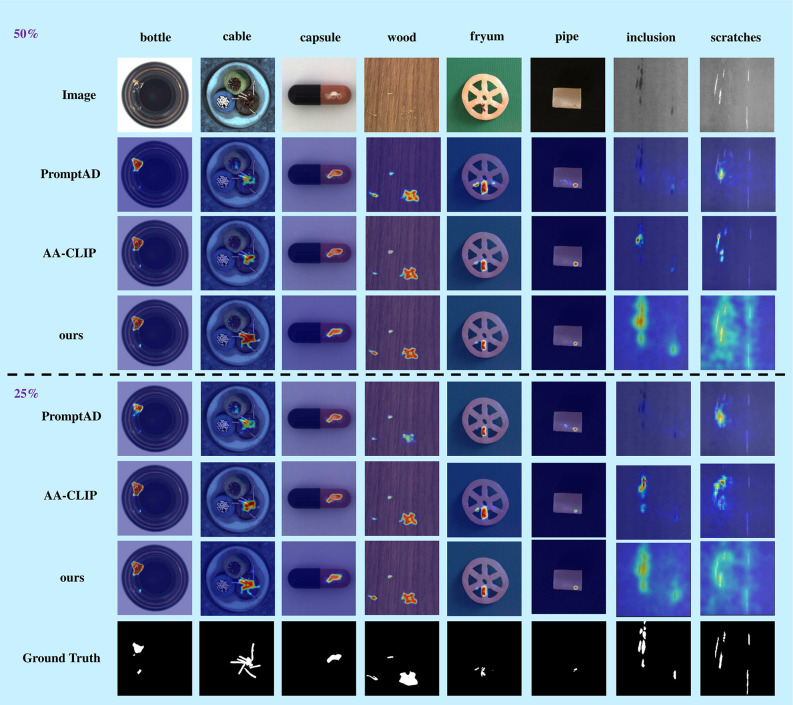
Qualitative comparison of anomaly localization maps under image down-scaling.

**Fig 6:**
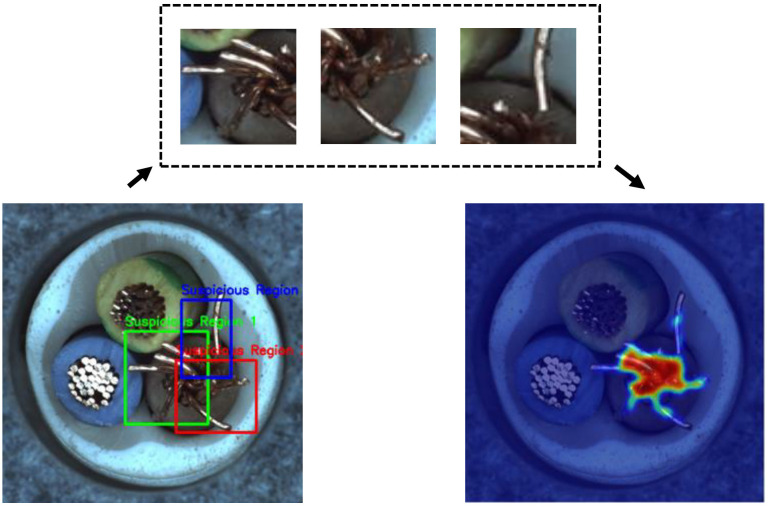
Visualization of the super-resolution zooming mechanism on a 50%scaled image.

As shown in the qualitative results in Fig 5, the proposed method demonstrates superior capability in detecting subtle anomalies under significant image down-scaling. Compared to baseline models, our approach generates anomaly localization maps with markedly higher spatial accuracy and stronger suppression of background noise. This performance is attributed to our super-resolution zooming mechanism, as shown in the second column of Fig 6. For challenging low-resolution inputs where subtle defects become nearly imperceptible, our method actively localizes suspicious regions and applies generative super-resolution to reconstruct high-fidelity, magnified image patches. This reconstruction effectively recovers critical structural and textural details essential for accurate defect identification, as evidenced by the clear revelation of defect patterns in the magnified views. These enhanced patches provide subsequent vision-language models with semantically rich and discriminative visual features, thereby enabling precise anomaly recognition and localization.

This study demonstrates that the introduced magnification mechanism significantly enhances sensitivity to subtle defects by enabling the targeted localization and reconstruction of suspicious regions. This strategy not only mitigates information loss from image down-scaling, but also improves robust recognition of low-contrast and small-target anomalies, thereby maintaining strong detection performance under challenging imaging conditions.

**Fig 7:**
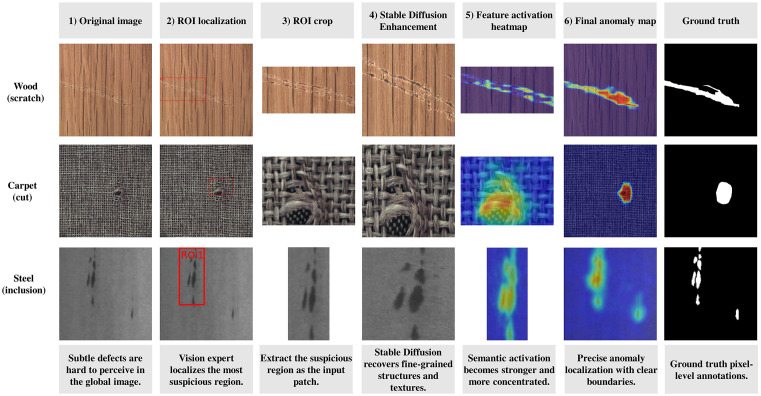
Progressive enhancement of subtle anomalies through ROI-guided perception. The suspicious region identified by the vision expert is selectively enhanced using Stable Diffusion, resulting in stronger anomaly-related feature responses and more precise localization of subtle defects.

To further investigate the mechanism by which the proposed framework improves anomaly detection, we visualize the progressive enhancement of subtle anomalies in Fig 7. The vision expert first identifies the most suspicious region in the image, which is then enhanced using Stable Diffusion to recover fine-grained structural and texture details within the ROI. This enhancement strengthens the local anomaly-related feature responses, resulting in more precise anomaly localization.

As illustrated in Fig 7, subtle defects in the original image are difficult to distinguish from normal regions due to low contrast and weak structural cues. After ROI localization, the framework concentrates on the most relevant region while suppressing background information. The subsequent enhancement of local features increases their discriminative capability, leading to stronger responses around defect regions. Consequently, the final anomaly maps exhibit clearer boundaries and more accurate localization. These observations indicate that the proposed framework improves anomaly detection performance by progressively amplifying subtle anomaly features rather than relying solely on stronger pre-trained feature extractors.

### 5.4 Ablation studies

#### 5.4.1 Step wise ablation on core components.

To quantitatively evaluate the contribution of each component in the proposed framework, comprehensive ablation experiments were conducted on the MVTec-AD, VisA, and NEU datasets under the 4-shot setting. The AA CLIP vision expert was adopted as the baseline model, and ROI extraction, multi scale fusion, and super resolution enhancement were progressively incorporated into the framework.

As reported in Table 5, introducing the vision expert guided ROI extraction leads to consistent performance improvements across all three datasets. The pixel level AUROC increases from 96.5% to 97.1% on MVTec-AD, from 97.4% to 97.6% on VisA, and from 96.3% to 96.5% on NEU. These results indicate that restricting feature extraction to the most informative regions reduces the influence of irrelevant background content and facilitates the detection of subtle defects.

**Table 5:** Step-wise ablation results on three benchmark datasets.

ROI	Fusion	SR	MVTec-AD	VisA	NEU
×	×	×	96.5 ± 0.2	97.4 ± 0.3	96.3 ± 0.2
√	×	×	97.1 ± 0.3	97.6 ± 0.4	96.5 ± 0.4
√	√	×	97.8 ± 0.2	97.9 ± 0.3	96.8 ± 0.4
√	√	Bicubic	98.2 ± 0.1	97.9 ± 0.1	96.9 ± 0.3
√	√	SD	98.6 ± 0.1	98.1 ± 0.1	97.2 ± 0.1

Further improvements are obtained after incorporating the proposed multi scale fusion module. Compared with the ROI based configuration, the pixel level AUROC increases from 97.1% to 97.8% on MVTec-AD, from 97.6% to 97.9% on VisA, and from 96.5% to 96.8% on NEU. This observation suggests that aggregating anomaly cues from multiple feature scales enables a more comprehensive representation of defect characteristics and improves localization accuracy.

To evaluate the effectiveness of image enhancement, bicubic interpolation and Stable Diffusion based super resolution were further investigated. Compared with the configuration without image enhancement, bicubic interpolation improves the pixel level AUROC to 98.2%, 97.9%, and 96.9% on MVTec-AD, VisA, and NEU, respectively. Replacing bicubic interpolation with Stable Diffusion based enhancement yields additional gains, resulting in final AUROC values of 98.6% on MVTec-AD, 98.1% on VisA, and 97.2% on NEU. The improvements are particularly evident on MVTec-AD and VisA, suggesting that enhancing fine grained structural details within suspicious regions facilitates more accurate anomaly localization.

Taken together, the results reveal a consistent performance improvement as each component is progressively incorporated into the framework. The full configuration achieves the highest performance on all three datasets, indicating that ROI extraction, multi scale fusion, and Stable Diffusion based enhancement provide complementary benefits and jointly contribute to the effectiveness of the proposed framework.

#### 5.4.2 Generalization across different vision experts.

To evaluate whether the observed performance gains originate from the proposed framework rather than from a specific vision expert, experiments were conducted using four representative anomaly detection experts, including WinCLIP, AdaCLIP, PatchCore, and AA-CLIP. These methods represent different anomaly detection paradigms and exhibit varying baseline performance levels. To provide a controlled comparison across different experts, the experiments were conducted on the MVTec-AD dataset under the 4-shot setting using Pixel-AUROC as the evaluation metric, since this benchmark is the most widely adopted dataset for industrial anomaly detection and contains diverse defect categories.

**Fig 8:**
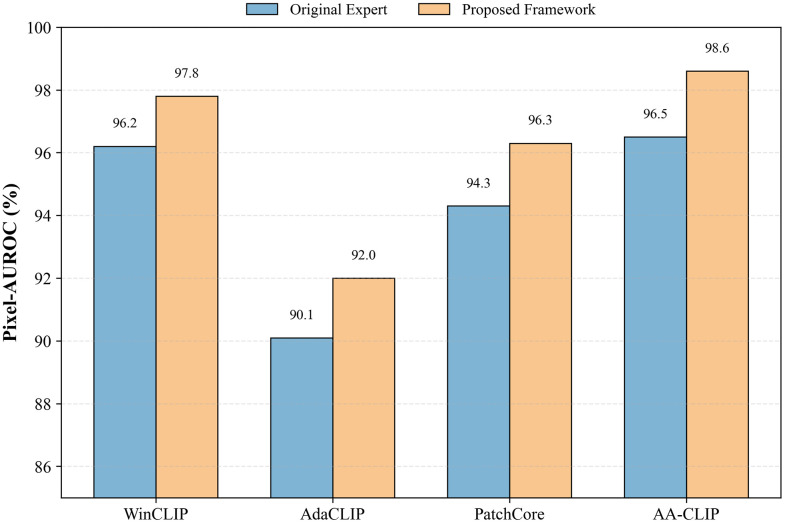
Performance comparison of different vision experts before and after integrating the proposed framework.

For each expert, the proposed framework was integrated without modifying the original model architecture. The detection performance before and after incorporating the framework is summarized in Table 6 and illustrated in Fig 8.

**Table 6:** Performance gains achieved by integrating the proposed framework with different vision experts on MVTec-AD.

Vision Expert	Original Expert	Framework	Improvement
WinCLIP	96.2	97.8	+1.6
AdaCLIP	90.1	92.0	+1.9
PatchCore	94.3	96.3	+2.0
AA-CLIP	96.5	98.6	+2.1

As shown in Table 6, consistent improvements are observed across all evaluated experts. Specifically, the proposed framework improves the Pixel-AUROC from 96.2% to 97.8% for WinCLIP, from 90.1% to 92.0% for AdaCLIP, from 94.3% to 96.3% for PatchCore, and from 96.5% to 98.6% for AA-CLIP. The corresponding gains range from 1.6 to 2.1 percentage points. Notably, even AA-CLIP, which already provides strong baseline performance, achieves a further improvement of 2.1%.

Fig 8 provides a visual comparison of the performance improvements achieved by different experts after integrating the proposed framework. Despite the differences in their original performance levels, all experts benefit from the framework and exhibit consistent performance gains.

Overall, the results demonstrate that the effectiveness of the proposed framework is not limited to a particular expert architecture. The framework can be readily integrated with different anomaly detection experts and consistently improve detection performance across models with different architectures and baseline capabilities. Therefore, the observed gains cannot be solely attributed to the strength of a particular vision expert.

#### 5.4.3 Impact on the multi-stage features.

**Fig 9:**
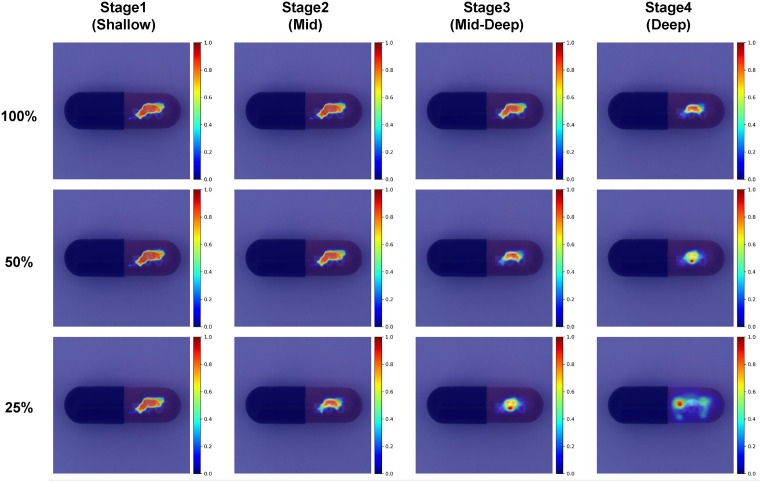
Visualization results of heat maps at four stages from the multi-scale ROI fusion module.

To evaluate the effectiveness of the multi-stage feature fusion mechanism, we visualized the multi-level features extracted by the proposed method at different image scales. Fig 9 displays the multi-scale feature response maps obtained from the four stages of the image encoder, corresponding to input images at 100%, 50%, and 25% of the original size.

Observations indicate that the model progressively localizes anomalous regions while effectively suppressing background interference and irrelevant areas across various scales. In deeper layers, feature responses become increasingly concentrated at actual defect locations, demonstrating the strong semantic selectivity and structural awareness of the multi-scale ROI fusion module. Even as image size decreases, the model maintains well-localized responses within suspicious regions, and the extracted patch features remain highly discriminative across resolutions, confirming the robustness of the method to scale variations.

**Fig 10:**
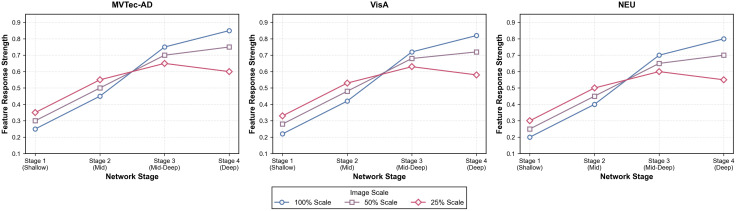
Hierarchical feature response patterns under multi-scale conditions.

Line plots depict feature response strength at four network stages (Stage 1–4) under different image resolutions (100%, 50%, 25% scales) for MVTec-AD, VisA, and NEU datasets, as shown in Fig 10. Feature response strength across four encoder stages (shallow to deep) evaluated at three image scales. All datasets exhibit similar trends: shallow layers (Stage 1–2) show increased relative importance at lower resolutions, while deep layers (Stage 3–4) preserve strong semantic representations across scale variations.

These results underscore the critical role of the multi-stage feature fusion mechanism in enhancing the model’s semantic understanding. This process leverages shallow features to preserve detailed spatial information and deep features to supply high-level semantic abstractions. Through cross-stage integration and region-aware alignment, the model achieves comprehensive anomaly perception spanning local to global patterns. Even under substantial image resolution reduction, the model sustains precise anomaly localization and recognition performance.

#### 5.4.4 Effectiveness evaluation of Stable Diffusion for super-resolution.

To assess the impact of super-resolution methods on anomaly detection, we conducted comprehensive experiments across all 15 categories of the MVTec AD dataset. Three ROI enhancement strategies were evaluated: Stable Diffusion, bicubic interpolation, and direct scaling without super-resolution, with Stable Diffusion implemented using the stable-diffusion-x4-upscaler configuration. As summarized in Table 7, Stable Diffusion outperforms bicubic interpolation in 14 categories, achieving an average pixel-level AUROC of 93.07% compared to 89.86%, corresponding to an overall improvement of approximately 3.2 percentage points. The only exception was the transistor category, which showed a marginal decrease of 0.83 percentage points, likely due to random variation.

**Table 7:** Comparison of pixel-level AUROC for Stable Diffusion, bicubic, and no upscaling on MVTec AD.

Category	SD	Bicubic	None
bottle	86.7	85.7	84.9
cable	82.4	81.2	72.3
capsule	93.1	83.8	81.7
carpet	97.3	96.6	95.6
grid	95.5	89.8	89.4
hazelnut	97.6	91.0	89.3
leather	91.3	89.4	89.1
metal_nut	93.0	91.5	84.9
pill	90.3	88.9	84.1
screw	98.0	87.9	82.6
tile	97.0	95.7	94.7
toothbrush	96.4	94.4	93.1
transistor	83.5	84.3	82.6
wood	97.4	91.6	87.4
zipper	96.7	96.4	96.2

A paired t-test across the 15 AUROC categories further confirmed the statistical significance of this improvement. Stable Diffusion significantly outperformed bicubic interpolation (t = 3.92, p = 0.0015), indicating that the observed gains are robust and unlikely to result from chance.

**Fig 11:**
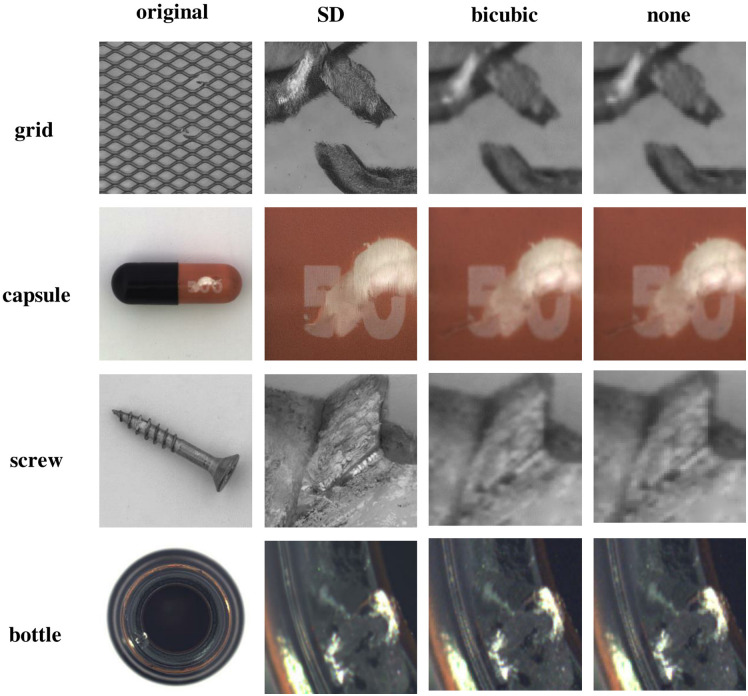
Visual comparison of ROI reconstruction quality using different super-resolution methods.

Visual inspection of reconstructed defective regions provides additional insight. Fig 11 presents ROI images from four representative categories: grids, capsules, screws, and bottles. For grids, Stable Diffusion produces noticeably sharper edges, while bicubic interpolation introduces blurring. Subtle scratches in capsules become clearly visible with Stable Diffusion enhancement. Fine screw threads are effectively restored, preserving high-frequency details lost in bicubic interpolation. In contrast, bottles, with smooth surfaces and large defect regions, exhibit minimal visual difference between the methods, consistent with a modest AUROC improvement of only one percentage point.

These results illustrate the applicability pattern of Stable Diffusion for super-resolution. It excels at recovering high-frequency details and regular textures, yielding pronounced performance gains in categories with rich textures or fine structural patterns, such as grids, screws, capsules, hazelnuts, and wood. For categories with smooth surfaces or extensive defects, such as bottles and transistors, conventional interpolation suffices, and the incremental benefits of Stable Diffusion are limited.

Finally, we evaluated Stable Diffusion in an industrially relevant scenario: detecting surface defects on rolled steel. Hot-rolled plates typically exhibit directional textures along the rolling direction, with complex patterns such as scale, scratches, and plaques, representing fine-grained structures rather than smooth surfaces. Accordingly, Stable Diffusion is theoretically well-suited for detecting rolling defects. Combining quantitative metrics and visual observations, we demonstrate that it substantially enhances industrial anomaly detection performance, particularly for subtle defects and textured surfaces. The framework allows flexible deployment, enabling selective use of Stable Diffusion based on specific industrial requirements, and its generalization can be further improved through domain-adaptive fine-tuning.

### 5.5 Practical deployment and efficiency analysis

While the previous sections focus on detection accuracy and generalization across experts, practical industrial deployment also requires consideration of computational efficiency and inference latency. To assess the applicability of the proposed framework in real-world scenarios, we analyzed the computational overhead of its components.

The average inference time for a 1024 × 1024 image is 3.5 s, with Stable Diffusion-based super-resolution accounting for approximately 2.8 s. In comparison, the vision expert-guided ROI extraction and multi-scale fusion modules introduce only minor overhead, indicating that the super-resolution stage represents the primary computational bottleneck.

To evaluate the tradeoff between efficiency and performance, we also investigated alternative lightweight enhancement strategies, including bicubic interpolation and direct scaling without super-resolution. Results show that bicubic interpolation substantially reduces inference time while still providing notable improvements over direct scaling. Although Stable Diffusion achieves the highest pixel-level AUROC, bicubic interpolation offers a more favorable balance between efficiency and detection performance.

These results indicate that the proposed framework can be flexibly deployed according to industrial requirements. In high-precision inspection scenarios, such as semiconductor manufacturing or fine-grained steel surface inspection, Stable Diffusion enhancement provides superior subtle defect perception. For latency-sensitive environments, lightweight strategies like bicubic interpolation can be adopted to achieve efficient yet effective anomaly detection.

## 6. Conclusions

This paper presents an industrial anomaly detection method based on vision-expert guidance and multi-scale feature fusion. The approach begins by localizing suspicious regions using a visual expert model, then enhances the representation of subtle defects through generative super-resolution to magnify and reconstruct these areas. A multi-stage feature fusion module based on a star graph attention mechanism is introduced to effectively integrate semantic information from both global images and multiple local regions. This module preserves contextual relationships while emphasizing high-fidelity details of anomalous areas. Experimental results demonstrate that the proposed method outperforms existing mainstream approaches across multiple datasets and few-shot settings, particularly in detecting subtle defects and generalizing across domains.

Although the proposed method achieves promising performance, several limitations remain. First, the quality of the visual expert model significantly affects the accuracy of suspicious-region extraction. If the expert model exhibits limited discriminative ability in specific categories, the effectiveness of subsequent processing may be compromised. Second, the computational overhead of super-resolution reconstruction and multi-scale feature fusion remains relatively high, which could pose challenges for deployment in real-time industrial applications. Additionally, the current method has not yet incorporated a dynamic text prompting mechanism, limiting its adaptability to previously unseen defect categories.

Future research will explore several promising directions to advance this work. Efforts will focus on developing lightweight feature extraction and fusion strategies to improve computational efficiency, alongside the investigation of adaptive prompt learning mechanisms to enhance zero-shot recognition of unseen anomaly types. The framework will also be extended to video anomaly detection and multi-modal industrial data scenarios, thereby broadening its practical applicability and generalization.
